# Non-Biomarker Bedside Risk Scores for One-Year Mortality After Acute Heart Failure Hospitalization

**DOI:** 10.14740/cr2241

**Published:** 2026-07-17

**Authors:** Duc Khanh Nguyen, Thanh Tuan Tran, Cao Cuong Tran

**Affiliations:** a School of Medicine, University of Medicine and Pharmacy at Ho Chi Minh City, Ho Chi Minh City, Vietnam

**Keywords:** Acute heart failure, Post-discharge follow-up, Mortality, Bedside risk score, Prognosis, Risk stratification, Real-world data

## Abstract

**Background:**

Simple bedside scores may support risk stratification after acute heart failure hospitalization, particularly during post-discharge follow-up when biomarker testing or complete registry-derived variables may be unavailable. We evaluated non-biomarker bedside risk scores for 1-year all-cause mortality after acute heart failure hospitalization in Vietnam.

**Methods:**

We conducted a retrospective cohort study of 497 adults hospitalized for acute heart failure at Cho Ray Hospital, Vietnam, between January and August 2021, with post-discharge outpatient follow-up and 1-year vital status ascertainment. This was a pragmatic evaluation of simplified bedside/domain scores constructed from routine clinical variables, not a formal external validation of the original GWTG-HF (Get With The Guidelines-Heart Failure) or OPTIMIZE-HF (Organized Program to Initiate Lifesaving Treatment in Hospitalized Patients With Heart Failure) equations. The primary score was an a priori-expanded eight-item, non-biomarker score including age, New York Heart Association class, systolic blood pressure, heart rate, serum sodium, serum creatinine, left ventricular ejection fraction, and hemoglobin. Natriuretic peptides and the AHEAD (atrial fibrillation, anemia, age, renal dysfunction and diabetes mellitus) score were not included. Discrimination was assessed using area under the receiver operating characteristic curve with bootstrap 95% confidence intervals, threshold characteristics and observed mortality across score groups.

**Results:**

Among 497 patients, 57 died within 1 year (11.5%). Point-score areas under the curve ranged from 0.525 to 0.590. The expanded eight-item score had an area under the curve of 0.590 (95% confidence interval, 0.509–0.660). At 4 or more points, sensitivity was 0.491, specificity was 0.655, positive likelihood ratio was 1.42, and negative likelihood ratio was 0.78. Observed mortality increased from 7.1% (95% confidence interval, 4.3–11.6) in the low-score group to 12.4% (7.7–19.4) in the intermediate-score group, and 15.6% (11.0–21.6) in the high-score group. A cross-validated clinical model using the same non-biomarker information achieved an area under the curve of 0.671.

**Conclusions:**

In this Vietnamese cohort after acute heart failure hospitalization, characterized by predominantly reduced or mildly reduced left ventricular ejection fraction, simple non-biomarker point scores provided modest prognostic information for 1-year mortality. Their role is broad orientation rather than definitive individual prognostication, and external validation is needed before implementation.

## Introduction

Acute heart failure is a frequent cause of unplanned hospital admission and is associated with substantial short- and long-term mortality. Contemporary heart failure guidelines emphasize early diagnosis, risk assessment, structured post-discharge management and outpatient follow-up [1, 2]. At the same time, hospitalized heart failure continues to impose a major global clinical and economic burden [3]. Early prognostic assessment after acute heart failure hospitalization and during post-discharge follow-up can support follow-up intensity, monitoring, therapy optimization and communication of prognosis.

Several prognostic tools for hospitalized heart failure were developed from large registries. The Acute Decompensated Heart Failure National Registry (ADHERE) classification and regression tree highlighted blood urea nitrogen, systolic blood pressure and serum creatinine for in-hospital mortality [4]. The OPTIMIZE-HF (Organized Program to Initiate Lifesaving Treatment in Hospitalized Patients With Heart Failure) and GWTG-HF (Get With The Guidelines-Heart Failure) models similarly emphasized age, hemodynamics and renal function [5, 6]. These tools are clinically important, but their exact variables, weights and intended outcomes are not always transportable to a local retrospective dataset that was not prospectively designed for score calculation.

In many real-world hospitals and post-discharge clinics, especially in resource-variable settings, a complete registry-derived dataset may not be collected in a standardized way. This creates a practical need to understand how far non-biomarker bedside approaches based on commonly available clinical variables can support preliminary risk stratification. Such variables include age, symptoms, blood pressure, heart rate, routine chemistry, hemoglobin and echocardiographic systolic function.

This study was designed to provide a conservative and methodologically transparent evaluation of non-biomarker bedside risk stratification for 1-year mortality among Vietnamese patients hospitalized for acute heart failure at Cho Ray Hospital and subsequently followed after discharge. The study deliberately addressed a narrow operational question: how well can simple, routine-care, non-biomarker scores stratify risk when natriuretic peptide-centered analyses or comorbidity-score analyses are not the focus? The GWTG-HF– and OPTIMIZE-HF–inspired domain scores were evaluated as pragmatic simplified approximations, not as formal external validations of the original registry-derived weighted equations.

## Materials and Methods

### Study design and setting

We performed a retrospective observational cohort study using routine hospitalization records and linked post-discharge follow-up documentation from adults hospitalized for acute heart failure at Cho Ray Hospital, a tertiary care hospital in Ho Chi Minh City, Vietnam. Patients were identified from index acute heart failure hospitalizations occurring between January 2021 and August 2021, with 1-year vital status assessed after the index hospitalization. The report was prepared with attention to the Strengthening the Reporting of Observational Studies in Epidemiology (STROBE) and the Transparent Reporting of a Multivariable Prediction Model for Individual Prognosis Or Diagnosis (TRIPOD) principles where applicable [7, 8].

### Participants

Adults with a primary clinical diagnosis of acute heart failure during the index hospitalization were eligible. Patients were excluded if acute heart failure was not the dominant clinical syndrome in the linked index episode or if primary outcome status was unavailable. The final analytic cohort comprised all 497 eligible patients in the locked deidentified analytic dataset. The variables used in the primary bedside score and benchmark model were complete in the analytic cohort and were abstracted from routine hospitalization records and linked follow-up documentation. Although left ventricular ejection fraction was complete and low on average, the dataset did not contain prospectively adjudicated heart failure with reduced ejection fraction (HFrEF), heart failure with mildly reduced ejection fraction (HFmrEF), and heart failure with preserved ejection fraction (HFpEF) phenotype labels; therefore, generalizability to patients with preserved ejection fraction should be cautious.

### Primary outcome

The primary outcome was 1-year all-cause mortality after the index acute heart failure hospitalization. This endpoint was selected because 1-year mortality is clinically relevant to post-discharge management, captures longer-term risk after an acute heart failure episode, and is less vulnerable than cause-specific mortality to misclassification in retrospective routine-care data. Vital status was determined from hospital records, scheduled and unscheduled outpatient follow-up documentation, and/or direct follow-up information recorded in the clinical database. When patients had no subsequent visit documented, the available direct follow-up information in the database was reviewed. Deaths were recorded as all-cause deaths; no adjudication of cause-specific mortality was attempted. Patients without ascertainable 1-year vital status were excluded. One-year vital status was available for all patients in the analytic cohort. Patients who were alive at 1 year were classified as survivors for the binary 1-year outcome. Because this was a retrospective routine-care study, no separate study-specific standardized follow-up protocol was implemented; however, available scheduled and unscheduled follow-up documentation and direct follow-up fields in the clinical database were reviewed uniformly for all eligible patients.

### Primary analysis and score construction

The primary analysis evaluated the performance of an a priori-defined expanded eight-item non-biomarker bedside score for 1-year all-cause mortality. The score assigned one point each for age 75 years or older, New York Heart Association class III–IV, systolic blood pressure 100 mm Hg or lower, heart rate 100 beats/min or higher, serum sodium lower than 135 mmol/L, serum creatinine 1.5 mg/dL or higher, left ventricular ejection fraction 30% or lower, and hemoglobin lower than 110 g/L. This score was selected as the primary score because it used the broadest set of routinely available non-biomarker domains in the dataset. These thresholds were selected before performance estimation based on commonly used clinically interpretable cut-points and registry-derived bedside domains, rather than optimized from the present outcome data.

### Biomarker restriction

Natriuretic peptide values were not used in the primary score construction, primary discrimination analysis or primary calibration analysis. This analytic restriction was applied to maintain a distinct non-biomarker bedside question and to avoid repeating biomarker-centered or comorbidity-score prognostic analyses.

### Secondary point scores

Additional non-biomarker point scores were evaluated as secondary comparators, including a hemodynamic-renal four-item score, an OPTIMIZE-HF–inspired domain score, a GWTG-HF–inspired domain score, and a routine bedside seven-item score. These inspired scores were not intended to reproduce or externally validate the original registry-derived weighted equations. Instead, they were transparent domain approximations constructed from available bedside variables.

### Routine-care clinical model benchmark

Because unweighted point scores may underuse available information, we additionally fitted a routine-care clinical benchmark model using non-biomarker variables only, including age, sex, systolic blood pressure, heart rate, New York Heart Association class, hemoglobin, creatinine, sodium, left ventricular ejection fraction, atrial fibrillation, diabetes mellitus, renal disease, and ischemic heart disease. An L2-regularized logistic regression model was used, and five-fold stratified cross-validation with a fixed random seed was used to estimate out-of-fold predicted probabilities.

### Statistical analysis

Continuous variables are summarized as mean ± standard deviation or median (interquartile range), and categorical variables as counts and percentages. Between-group comparisons were performed using Welch *t* test, Mann-Whitney U test, Chi-square test or Fisher exact test, as appropriate. Discrimination of each point score was assessed using the area under the receiver operating characteristic curve for 1-year all-cause mortality. Bootstrap 95% confidence intervals for areas under the curve were calculated using 1,000 resamples. Operational performance at pragmatic thresholds was described using sensitivity, specificity, positive and negative likelihood ratios, positive predictive value and negative predictive value. The primary calibration assessment compared observed 1-year mortality across low (0–2 points), intermediate (3 points) and high (4 points or more) groups of the expanded non-biomarker score. Wilson 95% confidence intervals were added for observed mortality in each score group, and absolute risk differences and risk ratios between groups were described as additional grouped calibration summaries. Because the point score does not generate calibrated individual predicted probabilities, calibration was assessed descriptively rather than by estimating calibration intercept and slope. Model performance was interpreted using established principles for prediction model assessment [9–12]. The cross-validated routine-care clinical model was reported as an exploratory benchmark rather than a replacement for bedside scoring. Analyses were performed using Python 3.11 with pandas, NumPy, SciPy, and scikit-learn. Two-sided P values < 0.05 were considered statistically significant for descriptive comparisons.

All variables used in the primary score and benchmark model were complete in the analytic dataset; therefore, no imputation was performed. To limit data-driven optimism, score definitions and clinically pragmatic thresholds were specified before performance estimation, and model benchmarking used out-of-fold predictions from cross-validation.

### Ethics

The study was approved by the Ethics Committee in Biomedical Research, University of Medicine and Pharmacy at Ho Chi Minh City (Approval No. 953/HDDD-DHYD; October 16, 2023). The requirement for informed consent was waived because this was a retrospective analysis of deidentified data collected during routine clinical care. The study was conducted in compliance with the ethical standards of the responsible institution and with the Declaration of Helsinki.

## Results

### Patient characteristics

The final analytic cohort included 497 patients hospitalized for acute heart failure at Cho Ray Hospital between January 2021 and August 2021, with subsequent post-discharge follow-up and 1-year vital status ascertainment. The mean age was 69.8 ± 15.2 years, and 284 patients (57.1%) were men. During 1 year of follow-up, 57 patients died (11.5%). Patients who died were older and had lower hemoglobin levels than those who survived. Mean left ventricular ejection fraction was 33.0±5.9%, indicating a cohort dominated by reduced or mildly reduced systolic function rather than a broadly balanced HFrEF/HFmrEF/HFpEF sample; prospectively adjudicated phenotype labels were not available in the locked dataset. Baseline characteristics are shown in Table 1.

**Table 1:** Baseline Characteristics Overall and by 1-Year Mortality Status

Characteristic	Overall (n = 497)	Survived 1 year (n = 440)	Died within 1 year (n = 57)	P value
Age, years	69.8 ± 15.2	69.3 ± 15.4	74.0 ± 13.5	0.018
Male sex, n (%)	284 (57.1%)	252 (57.3%)	32 (56.1%)	0.984
New York Heart Association class III–IV, n (%)	379 (76.3%)	337 (76.6%)	42 (73.7%)	0.749
Systolic blood pressure, mm Hg	108.9 ± 22.1	109.0 ± 22.2	108.7 ± 21.2	0.941
Heart rate, beats/min	97.3 ± 15.6	97.0 ± 15.5	100.0 ± 16.2	0.197
Hemoglobin, g/L	120.4 ± 23.0	122.0 ± 22.7	107.8 ± 21.5	< 0.001
Creatinine, mg/dL	1.27 (1.09–1.51)	1.26 (1.09–1.51)	1.31 (1.14–1.53)	0.191
Sodium, mmol/L	136.6 ± 4.9	136.7 ± 4.9	136.0 ± 5.0	0.333
Left ventricular ejection fraction, %	33.0 ± 5.9	33.2 ± 5.7	31.3 ± 6.7	0.044
Atrial fibrillation, n (%)	82 (16.5%)	73 (16.6%)	9 (15.8%)	1.000
Diabetes mellitus, n (%)	143 (28.8%)	119 (27.0%)	24 (42.1%)	0.027
Renal disease, n (%)	59 (11.9%)	52 (11.8%)	7 (12.3%)	1.000
Ischemic heart disease, n (%)	260 (52.3%)	233 (53.0%)	27 (47.4%)	0.513

### Non-biomarker bedside score definitions

The evaluated scores represented increasingly broad non-biomarker domains, from a hemodynamic-renal score to an expanded bedside score incorporating age, symptoms, hemodynamics, renal function, sodium, systolic function and hemoglobin. The OPTIMIZE-HF–inspired and GWTG-HF–inspired domain scores were intentionally described as simplified approximations rather than exact external validations of the original tools (Table 2).

**Table 2:** A Priori-Defined Non-Biomarker Bedside Score Definitions

Score	Definition	Range
Hemodynamic-renal four-item score	1 point each: systolic blood pressure ≤ 100 mm Hg; heart rate ≥ 100 beats/min; sodium < 135 mmol/L; creatinine ≥ 1.5 mg/dL.	0–4
OPTIMIZE-HF–inspired domain score	1 point each: age ≥ 75 years; systolic blood pressure ≤ 100 mm Hg; heart rate ≥ 100 beats/min; sodium < 135 mmol/L; creatinine ≥ 1.5 mg/dL; left ventricular ejection fraction ≤ 40%. This is an inspired domain-level approximation and not the original OPTIMIZE-HF weighted equation.	0–6
GWTG-HF–inspired domain score	1 point each: age ≥ 75 years; systolic blood pressure ≤ 110 mm Hg; heart rate ≥ 100 beats/min; sodium < 135 mmol/L; creatinine ≥ 1.5 mg/dL. Blood urea nitrogen, chronic obstructive pulmonary disease and race were intentionally not approximated; therefore, this is not the original GWTG-HF weighted equation.	0–5
Routine bedside seven-item score	1 point each: age ≥ 75 years; New York Heart Association class III–IV; systolic blood pressure ≤ 100 mmHg; heart rate ≥ 100 beats/min; sodium < 135 mmol/L; creatinine ≥ 1.5 mg/dL; left ventricular ejection fraction ≤ 30%.	0–7
Expanded non-biomarker eight-item score (primary score)	Routine bedside seven-item score plus low hemoglobin (hemoglobin < 110 g/L).	0–8
Cross-validated routine-care clinical model	L2-regularized logistic regression using non-biomarker routine-care variables: age, sex, systolic blood pressure, heart rate, New York Heart Association class, hemoglobin, creatinine, sodium, left ventricular ejection fraction, atrial fibrillation, diabetes mellitus, renal disease, and ischemic heart disease. Five-fold stratified cross-validation was used.	Probability

GWTG-HF: Get With The Guidelines-Heart Failure; OPTIMIZE-HF: Organized Program to Initiate Lifesaving Treatment in Hospitalized Patients With Heart Failure.

### Discrimination and operational performance

**Figure 1:**
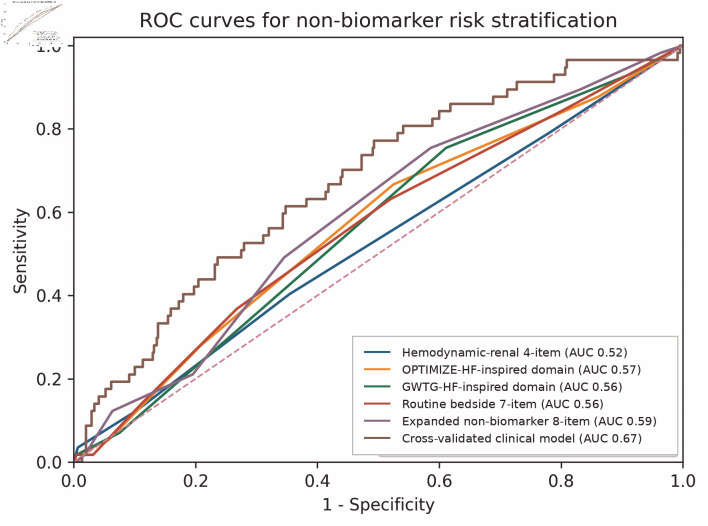
Receiver operating characteristic curves for non-biomarker bedside scores and the cross-validated routine-care clinical model. ROC curves are shown for five point scores and the cross-validated routine-care clinical model, including OPTIMIZE-HF–inspired and GWTG-HF–inspired domain scores that were not formal external validations of the original weighted equations. No natriuretic peptide value or AHEAD score was included in the primary analyses. AHEAD: atrial fibrillation, anemia, age, renal dysfunction and diabetes mellitus; AUC: area under the receiver operating characteristic curve; CV: cross-validated; GWTG-HF: Get With The Guidelines-Heart Failure; OPTIMIZE-HF: Organized Program to Initiate Lifesaving Treatment in Hospitalized Patients With Heart Failure; ROC: receiver operating characteristic.

**Figure 2:**
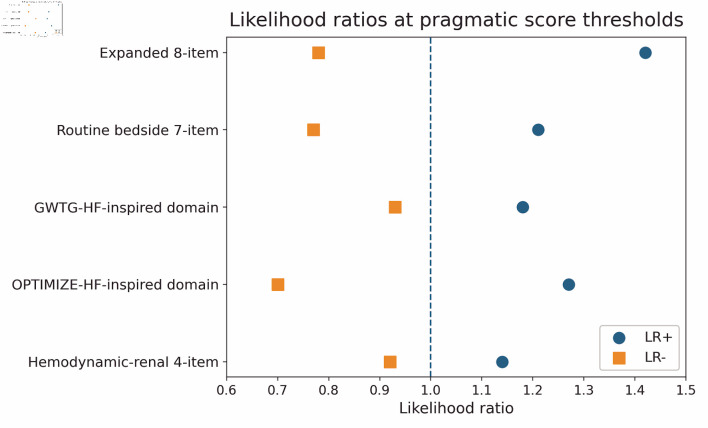
Likelihood ratios at pragmatic score thresholds. Likelihood ratios were modest across the evaluated non-biomarker point scores, including the inspired domain scores, indicating that these scores should not be used as standalone definitive prognostic instruments. LR: likelihood ratio; GWTG-HF: Get With The Guidelines-Heart Failure; OPTIMIZE-HF: Organized Program to Initiate Lifesaving Treatment in Hospitalized Patients With Heart Failure.

Discrimination of simple non-biomarker point scores was modest. The hemodynamic-renal four-item score had the lowest area under the curve (0.525), whereas the expanded non-biomarker eight-item score had the highest point-score area under the curve (0.590; 95% confidence interval, 0.509–0.660). The cross-validated routine-care clinical model using the same non-biomarker information had better discrimination (area under the curve, 0.671; 95% confidence interval, 0.597–0.743). Operational characteristics at pragmatic thresholds are presented in Table 3 and Figure 1. Likelihood ratios were modest across all thresholds (Fig. 2).

**Table 3:** Discrimination and Operational Characteristics for 1-Year All-Cause Mortality

Score	AUC (bootstrap 95% CI)	Threshold	Sensitivity	Specificity	LR+	LR-	PPV	NPV
Hemodynamic-renal four-item	0.525 (0.446–0.600)	≥ 2	0.404	0.645	1.14	0.92	0.128	0.893
OPTIMIZE-HF–inspired domain	0.567 (0.492–0.639)	≥ 3	0.667	0.475	1.27	0.70	0.141	0.917
GWTG-HF–inspired domain	0.562 (0.489–0.630)	≥ 3	0.333	0.718	1.18	0.93	0.133	0.893
Routine bedside seven-item	0.563 (0.483–0.634)	≥ 3	0.632	0.480	1.21	0.77	0.136	0.909
Expanded non-biomarker eight-item	0.590 (0.509–0.660)	≥ 4	0.491	0.655	1.42	0.78	0.156	0.909

AUC: area under the receiver operating characteristic curve; CI: confidence interval; GWTG-HF: Get With The Guidelines-Heart Failure; OPTIMIZE-HF: Organized Program to Initiate Lifesaving Treatment in Hospitalized Patients With Heart Failure; LR: likelihood ratio; PPV: positive predictive value; NPV: negative predictive value.

### Simple calibration of the primary non-biomarker score

**Figure 3:**
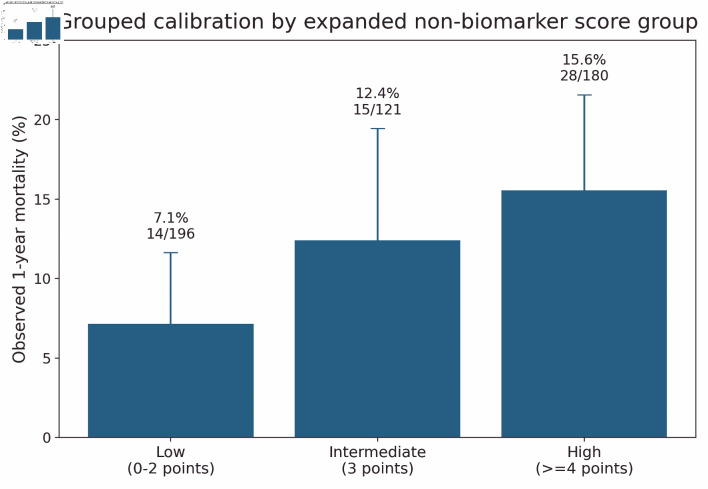
Grouped calibration of observed 1-year mortality by expanded non-biomarker score group. Observed 1-year all-cause mortality with Wilson 95% confidence intervals is shown across low, intermediate and high categories of the primary expanded non-biomarker 8-item score.

The primary grouped calibration assessment showed a graded but limited separation in observed 1-year mortality across expanded non-biomarker score groups. Observed mortality was 7.1% (95% confidence interval, 4.3–11.6) in the low-score group, 12.4% (95% confidence interval, 7.7–19.4) in the intermediate-score group, and 15.6% (95% confidence interval, 11.0–21.6) in the high-score group (Table 4, Fig. 3). The absolute risk difference between the high- and low-score groups was 8.4 percentage points, and the crude risk ratio was 2.18 (95% confidence interval, 1.18–4.00). This pattern supports use of the score for broad initial orientation but not for precise individual risk prediction. As an exploratory benchmark, cross-validated predicted risk from the non-biomarker clinical model separated patients into tertiles, with observed 1-year mortality of 4.8%, 11.5%, and 18.1% in the low, intermediate, and high predicted-risk groups, respectively.

**Table 4:** Observed 1-Year Mortality by Expanded Non-Biomarker Eight-Item Score Group, With Wilson 95% CIs

Primary score group	N	Deaths	Observed 1-year mortality (Wilson 95% CI)
Low (0–2 points)	196	14	7.1% (4.3–11.6)
Intermediate (3 points)	121	15	12.4% (7.7–19.4)
High (≥ 4 points)	180	28	15.6% (11.0–21.6)

CI: confidence interval.

## Discussion

In this real-world single-center cohort of adults hospitalized for acute heart failure in Vietnam, with post-discharge follow-up and 1-year vital status ascertainment, non-biomarker bedside point scores provided only modest discrimination for 1-year all-cause mortality. The best-performing point score, an expanded eight-item non-biomarker score, achieved an area under the curve of 0.590 and showed a graded but limited separation in observed mortality across low, intermediate, and high score groups. A cross-validated model using the same routine-care non-biomarker information performed better, suggesting that clinically meaningful prognostic information was present but was partly lost when continuous variables were dichotomized and converted into simple equal-weight point scores.

Several factors likely explain the modest predictive performance of the simplified point scores. First, the historical GWTG-HF and OPTIMIZE-HF models used original variables, weights and coefficients that were developed primarily for in-hospital mortality, whereas this study evaluated 1-year all-cause mortality after the index hospitalization and post-discharge follow-up. Second, several original registry variables were unavailable or intentionally not approximated, and the bedside scores assigned equal weight to dichotomized variables. Third, differences in patient population, healthcare setting, follow-up structure and local practice patterns may reduce transportability of registry-derived domains across settings.

Our results are consistent with the broader experience of heart failure risk prediction. Large registry-derived tools such as the ADHERE, OPTIMIZE-HF and GWTG-HF models demonstrated the importance of hemodynamics, renal function and age, but the present study intentionally did not claim external validation of those original weighted equations [4–6]. Instead, it examined what can be learned from transparent non-biomarker domains that are available in routine care and post-discharge follow-up.

The study also highlights an implementation issue for hospitals and post-discharge clinics considering bedside prognostic tools. A risk score can be clinically attractive yet statistically limited if variables are incomplete, coefficients are not transported, continuous information is dichotomized, or the intended outcome differs from the original derivation setting. Therefore, non-biomarker scores should be viewed as an initial layer of risk stratification rather than as a replacement for comprehensive clinical assessment.

The analytic scope of this manuscript was deliberately restricted to non-biomarker bedside information. Natriuretic peptides were not included in the primary analyses. This allowed the study to address a distinct operational question: how far can routine non-biomarker information support risk stratification after acute heart failure hospitalization when biomarker-centered or specific comorbidity-score analyses are not the focus?

### Clinical implications

Non-biomarker bedside scores may support early clinical orientation after acute heart failure hospitalization and during post-discharge follow-up, especially when used to identify groups with lower observed mortality. They should not be used as the sole basis for triage, follow-up intensity, therapeutic decisions or communication of prognosis. A staged approach may be preferable: simple bedside scoring for immediate orientation, followed by fuller clinical assessment, laboratory review, imaging interpretation and structured follow-up planning.

### Strengths and limitations

Strengths include a real-world acute heart failure hospitalization cohort with linked post-discharge follow-up at a tertiary hospital, complete 1-year outcome classification, explicit primary outcome definition and a conservative analytic strategy that avoided biomarker-centered primary analyses. The addition of bootstrap confidence intervals for areas under the curve, Wilson confidence intervals for grouped observed mortality, and simple grouped calibration of the primary score improves interpretability. Limitations include the single-center retrospective design, lack of external validation, modest number of deaths, use of simplified inspired point scores rather than original weighted equations, and the predominantly reduced or mildly reduced left ventricular ejection fraction profile of the cohort. The locked deidentified dataset did not contain prospectively adjudicated HFrEF, HFmrEF and HFpEF labels, so phenotype-specific generalizability could not be fully assessed. Because a detailed pre-analytic screening log was not available, the exact number of initially screened index hospitalizations and linked follow-up records and the number excluded at each pre-analytic step could not be reconstructed. Therefore, generalizability to patients with preserved ejection fraction should be cautious. In addition, the cross-validated routine-care clinical model was included only as an exploratory benchmark, and the modest event count may affect the stability of multivariable model-based estimates. The findings should therefore be interpreted as hypothesis-generating and should be validated in independent cohorts.

### Conclusions

Among Vietnamese patients hospitalized for acute heart failure at Cho Ray Hospital and followed for 1 year after discharge, in a cohort characterized by predominantly reduced or mildly reduced left ventricular ejection fraction, simple non-biomarker bedside point scores showed limited discrimination for 1-year all-cause mortality. The expanded non-biomarker eight-item score provided a graded but modest increase in observed mortality across score groups. A cross-validated routine-care clinical model performed better, supporting the concept that routine non-biomarker variables contain prognostic information but require careful modelling and external validation before decision-tool implementation.

## Data Availability

The deidentified data and statistical code are not publicly deposited because of institutional data protection requirements for patient-level clinical data. They may be made available by the corresponding author upon reasonable request, subject to institutional approval and applicable privacy safeguards.

## Abbreviations

ADHERE: Acute Decompensated Heart Failure National Registry
AHEAD: atrial fibrillation, anemia, age, renal dysfunction and diabetes mellitus
AUC: area under the receiver operating characteristic curve
BUN: blood urea nitrogen
CI: confidence interval
COPD: chronic obstructive pulmonary disease
GWTG-HF: Get With The Guidelines-Heart Failure
HFmrEF: heart failure with mildly reduced ejection fraction
HFpEF: heart failure with preserved ejection fraction
HFrEF: heart failure with reduced ejection fraction
IQR: interquartile range
LR: likelihood ratio
LVEF: left ventricular ejection fraction
NPV: negative predictive value
NYHA: New York Heart Association
OPTIMIZE-HF: Organized Program to Initiate Lifesaving Treatment in Hospitalized Patients With Heart Failure
PPV: positive predictive value
ROC: receiver operating characteristic
SBP: systolic blood pressure
STROBE: Strengthening the Reporting of Observational Studies in Epidemiology
TRIPOD: Transparent Reporting of a multivariable prediction model for Individual Prognosis Or Diagnosis
